# Pulmonary hypertension in an adult with unilateral absence of left pulmonary artery

**DOI:** 10.1177/2050313X221127667

**Published:** 2022-09-29

**Authors:** Armen Ghazarian, Matthew King, Ned Premyodhin, Irmina Gradus-Pizlo

**Affiliations:** 1 Department of Medicine, School of Medicine, University of California, Irvine, Orange, CI, USA; 2 Division of Cardiology, School of Medicine, University of California, Irvine, Orange, CI, USA

**Keywords:** Unilateral absence of pulmonary artery

## Abstract

Unilateral absence of pulmonary artery is a rare congenital abnormality that occurs due to malformation of the sixth aortic arch during embryonic development. The clinical presentation of unilateral absence of pulmonary artery can vary based on age of diagnosis; however, in the adult population, it can present with a variety of manifestations including hemoptysis, recurrent pneumonia, and pulmonary hypertension or as an incidental finding. Diagnosis and management of unilateral absence of pulmonary artery remain a challenge. Here, we describe a case of a 37-year-old female with no known past medical history who presented with progressively worsening dyspnea and fatigue. She was incidentally found to have unilateral absence of pulmonary artery on computerized tomography angiography of the chest. Her imaging and physical exam demonstrated signs of volume overload and severe pulmonary hypertension. She received diuretics with good response and was discharged with referral to pulmonary hypertension clinic and eventual follow-up with right heart catheterization. In summary, we describe a rare congenital condition and highlight its diagnostic and therapeutic challenges.

## Introduction

Unilateral absence of pulmonary artery (UAPA) is a rare congenital abnormality that occurs due to malformation of the sixth aortic arch during embryonic development, resulting in proximal interruption of the pulmonary artery (PA).^1^ UAPA commonly presents in one of two ways. The first is diagnosed early in infancy with congestive cardiac failure or pulmonary hypertension (PH) and is usually associated with cardiovascular congenital abnormalities such tetralogy of Fallot, atrial septal defect, and truncus arteriosus.^2^ Conversely, UAPA can have a delayed presentation in adulthood as an isolated finding without associated cardiovascular defects.^2^ In adults, 13%–30% remain asymptomatic and are diagnosed incidentally on chest radiograph; however, some can present with hemoptysis, recurrent lung infections, and dyspnea on exertion due to PH.^3^ Literature regarding isolated UAPA is limited, making diagnostic and therapeutic plans challenging.^1,4^ Given its rarity, we present a case of a young female with UAPA and PH.

## Case report

A 37-year-old female presented to the emergency department for 1 year of progressive dyspnea that worsened within the month prior to presentation. She reported previously tolerating her symptoms but had developed fatigue and exertional dyspnea notably needing to take more breaks when ascending stairs. The dyspnea was at times associated with palpitations and substernal chest pain. She denied any history of syncope. She additionally denied any prior tobacco or illicit drug use; however, she did admit to recent methamphetamine use to combat fatigue associated with working multiple jobs. As a child, she reportedly had a murmur that resolved in infancy but had no family history of congenital heart disease. She works as teacher at a parochial school and lives with her mother and her two children.

**Figure 1.**
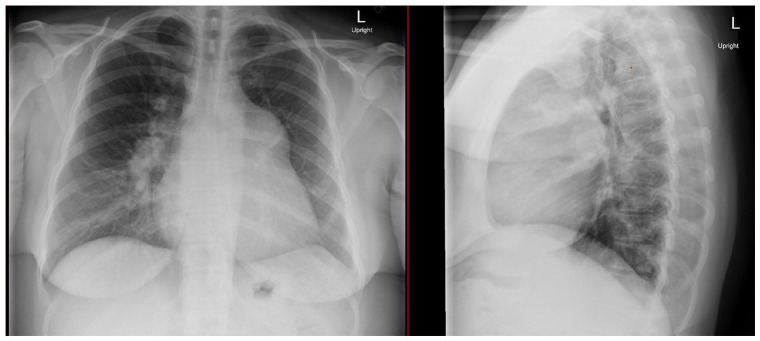
Chest X-ray demonstrating enlarged cardiac silhouette with mediastinal shift and large central pulmonary arteries.

Vital signs were notable for a heart rate of 90, blood pressure 108/92 mm Hg, and oxygen saturation of 99% on room air. She was afebrile. Her weight was 113.4 kg with body mass index (BMI) 39.15 kg/m^2^. On physical examination her jugular venous pulsation was elevated to 12 cm of water, heart was regular rate, normal S_1_, P2 louder then A_2_, palpable PA pulsation in the left second intercostal space, and grade III/VI diastolic murmur and grade III/VI systolic murmur over the left sternal border with right ventricular (RV) heave. She had trace bilateral lower extremity edema. Chest radiograph (CXR) showed an enlarged cardiac silhouette and large central pulmonary arteries (Figure 1). Electrocardiogram (ECG) showed normal sinus rhythm with right axis deviation, incomplete right bundle branch block, and RV hypertrophy. On admission, initial labs were significant for brain natriuretic peptide of 562 pg/mL (reference range = 0–100 pg/mL) and urine toxicology screen positive for amphetamines.

### Past medical history

The patient had no relevant past medical history. Surgical history was notable for cholecystectomy. She had history of two live vaginal births. Reportedly, she had 1-month history of methamphetamine abuse.

### Management

**Figure 2.**
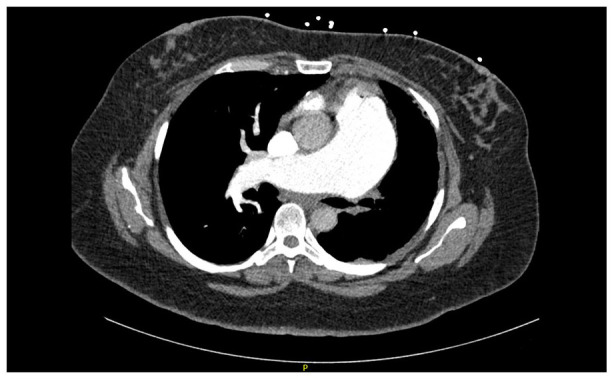
CT angiography chest showing agenesis of left branch of pulmonary artery.

An extensive work up had been performed prior to cardiology being consulted. Initial computerized tomography (CT) angiogram of the chest demonstrated left PA atresia with dilatation of the main and right pulmonary arteries. The main PA measured up to 5.9 cm in diameter. Findings of increased right heart size and reflux of contrast into the distal hepatic veins were suggestive of elevated right heart pressures. No filling defects were seen to suggest pulmonary embolism (Figure 2). Given the above findings, a transthoracic echocardiogram (TTE) was obtained that showed severe right atrial and ventricular dilatation with normal RV systolic function. There was severe tricuspid and pulmonic regurgitation with a severely elevated PA systolic pressure of 84.00 mm Hg. The left ventricle had normal systolic function with an ejection fraction of 62%. Saline contrast bubble study was negative. Rheumatologic serologies were negative. Given the patient had signs of volume overload and dyspnea, she underwent diuresis with furosemide until improvement in exam and symptoms. Following multidisciplinary review between pulmonology and cardiology, an inpatient right heart catheterization was not pursued given her clinical improvement, absence of high-risk symptoms such as chest pain and syncope, and the inherent elevated risk of perforation due to her complicated anatomy and profoundly enlarged pulmonary trunk. Upon discharge, she underwent a 6-min walk test with limited distance of 150 m and complaints of dyspnea on exertion; however, she did not desaturate below 92% on room air. Patient had outpatient referral set up with PH clinic.

Two months following indexed hospitalization, patient followed up with PH clinic and subsequently underwent outpatient lung ventilation/perfusion scintigraphy and right heart catheterization. Lung ventilation/perfusion scintigraphy revealed minimal to absent perfusion of the entire left lung; however, normal ventilation was maintained. Right lung was normal. The right heart catheterization revealed a mean right arterial pressure of 10 mm Hg, mean pulmonary artery pressure (mPAP) of 58 mm Hg, and PA wedge pressure of 7–10 mm Hg. The pulmonary vascular resistance (PVR) was 10 Wood units, the cardiac index was 2.2 L/min/m^2^, and partially positive vasoreactivity test with dropping of PVR >20% with higher dose of inhaled nitrous oxide. Patient will be started on vasodilatory treatment with a combined endothelin receptor antagonist (ERA) and phosphodiesterase-5 inhibitor (PDE5i) in addition to a trial of calcium channel blocker pending medical insurance preauthorization.

## Discussion

UAPA is a rare congenital abnormality that occurs due to malformation of the sixth aortic arch during embryonic development.^1^ It can occur as an isolated disorder presenting in adulthood or can be found in infancy typically associated with other cardiovascular abnormalities.^5^ Isolated UAPA has an estimated prevalence of 1 in 200,000 based on reviews.^6^ The diagnosis of UAPA is easy to miss and requires a thorough history, physical examination, and imaging. Physical exam can show asymmetric lung fields which can be confirmed with CXR. The mediastinum tends to be shifted toward the affected side with the absence of hilar vasculature.^1,2^ If suspicion is high enough, diagnosis can be made by CT, magnetic resonance imaging (MRI), or echocardiogram; however, the gold standard is pulmonary angiogram.^1,3^ ECG is usually normal unless there is PH, in which case it would demonstrate RV dominance.^1,2^ Our patient had a reported history of a childhood murmur that presented as a newborn and resolved in infancy; we believe that this murmur was likely due to a patent ductus arteriosus and likely resolved with closure.^1^ However, she could not give further detailed information.

Review of the literature returned few reported cases of isolated UAPA and PH.^4,7^ The incidence of PH among patients with UAPA has been reported to be between 20% and 44%.^2–6^ It is theorized that PH occurs from increased blood flow through the patent PA into one lung resulting in increased shear stress in the endothelium leading to injury.^1,7^ Endothelin is then released subsequently vasoconstricting the pulmonary arterioles. Chronic vasoconstriction leads to PA vascular remodeling, resulting in increased resistance and PH.^1,7^ The factors that determine which patients develop PH are unknown.^7^ In our patient, a right heart catheterization was deferred for outpatient given her clinical improvement, absence of high-risk symptoms such as chest pain and syncope, and the inherent elevated risk of perforation due to her complicated anatomy and profoundly enlarged pulmonary trunk. Upon further assessment and discussion with the patient in clinic, she elected to undergo right heart catheterization with results mentioned above. We believe that the etiology of her PH is multifactorial including but not limited to her UAPA, morbid obesity, and suspected sleep apnea, and was likely further exacerbated by her methamphetamine abuse, which we believe to be the major contributing factor. Given her history of daytime sleepiness and high likelihood of obstructive sleep apnea, a referral for sleep study was placed. In addition, she was referred to a dietician to address both her morbid obesity.

The overall mortality rate across all age groups is reported to be 7% largely from RV failure, respiratory failure, and massive pulmonary hemorrhage.^1,6^ Avoidance of high altitudes and pregnancy are encouraged in these patients as they can worsen symptoms or uncover underlying PH and UAPA.^6^ Treatment for PH in patients with isolated UAPA varies with age. In pediatric patients who have isolated UAPA and are asymptomatic, serial TTEs are used to monitor for the development of PH; however, this can also probably be applied to UAPA found in adult patients who are asymptomatic.^7^ Surgical treatment such as revascularization with aortopulmonary shunt creation, PA anastomosis, and novel hybrid techniques utilizing intraductal stents is indicated in childhood and feasible as PA branch rescue may result in normalization of pulmonary pressure and avoidance of lung transplant.^1,8^ However, in older adult patients, surgical options with revascularization are not feasible because the intrapulmonary arteries are largely fibrosed.^1,7^ Therefore, pharmacotherapy with vasodilators is largely pursued in patients with PH.^1,6,7^ Another possible modality of therapy for this patient with PH is lung transplant.^1^ Our patient is currently being followed in PH clinic with continued hope of successful abstinence from methamphetamine which may open the possibility of lung transplant in the future.

## Conclusion

In summary, our patient presented with the rare congenital condition UAPA as an adult. Patients are often symptomatic for a long period of time but not aware of the severity of their illness due to slow progression of their disease during their life. They are undiagnosed even when they are under medical care. Our patient had two pregnancies and two deliveries, and was not suspected to have a congenital vascular disease. Late presentation may be associated with severe complications such as PH. This case highlights the diagnostic challenges of UAPA, the consequences of delayed diagnosis, and the need for increased awareness of this condition.
